# Cultivation of Genetically Modified Soybeans Did Not Alter the Overall Structure of Rhizosphere Soil Microbial Communities

**DOI:** 10.3390/plants14030457

**Published:** 2025-02-04

**Authors:** Wenjing Shen, Laipan Liu, Zhixiang Fang, Li Zhang, Zhentao Ren, Qi Yu, Xin Yin, Biao Liu

**Affiliations:** Key Laboratory on Biodiversity and Biosafety of Nanjing Institute of Environmental Sciences, Ministry of Ecology and Environment, Nanjing 210042, China; swj@nies.org (W.S.); liulaipan@163.com (L.L.); zxfang23@126.com (Z.F.); huanghe70885@sina.com (L.Z.); rztkkk@163.com (Z.R.); 15968821722@163.com (Q.Y.); njfu_shin@163.com (X.Y.)

**Keywords:** genetically modified soybean, 16S and ITS rDNA sequencing, microbial communities

## Abstract

Herbicide-tolerant soybeans are the most extensively cultivated genetically modified (GM) crop globally. The effects of GM soybean and associated agronomic practices on soil microbial communities remain poorly understood. This study aimed to investigate the impact of planting GM soybeans with a glyphosate application on soil microbial diversity. The main bacterial and fungal community compositions (phylum level) were consistent for GM and non-GM soybeans. The alpha diversity analysis indicated that the bacterial Shannon index was significantly higher in GM rhizosphere soil during flowering compared to non-GM soil. There were no significant differences in the Shannon, Simpson, or ACE indices of the soil fungal communities between GM and non-GM soybeans in the same period. The PCoA analysis showed no significant differences in community structure between the GM and non-GM soybean soil for either fungi or bacteria during the same period. Although the relative abundance of *Bradyrhizobium* at the seedling stage was significantly lower in those GM than in those non-GM, it did not affect the final number of root nodules in either soybean type. The relative abundance of *Frankia* was significantly lower in GM rhizosphere soil during the seedling and flowering stages, whereas that of *Thelebolus* was significantly higher during flowering and pod filling. The abundance and ecological functions of these taxa warrant continuous monitoring.

## 1. Introduction

The global area of genetically modified (GM) crop cultivation reached 20.63 million hectares in 2023, which is 118 times larger than the 1.7 million hectares in 1996. GM soybeans accounted for 72.4% of the global soybean cropland, covering an area of 10.09 million hectares [1]. It is important to ensure safety for the promotion of GM crop cultivation [2,3]. It is crucial to consider the impact on the composition and function of soil microbial communities for environmental safety assessment [4,5]. Plant roots release various exudates into the rhizosphere, attracting various microorganisms (bacteria, fungi, and algae) colonized around the rhizosphere, thereby affecting the structure and ecological functions of rhizosphere microbial communities [6,7,8]. Nadarajah reported that differences in the composition and quantity of root exudates among plants can affect the composition and function of the rhizosphere soil microbial communities [9]. 5-enolpyruvylshiki mate-3-phosphate synthase (EPSPS) was the most common target gene for GM crops. The application of EPSPS on GM crops significantly enhances the efficiency and cost-effectiveness of weed management practices. GM cultivation and agronomic practices may influence the diversity and functions of soil microbial communities. Previous studies have reported varying results regarding the impact of transgenic plants on soil microbial communities, particularly with respect to rhizobacterial communities [10,11].

In recent years, the development of high-throughput sequencing technology has made the large-scale sequencing of soil microbial genes possible, providing rich information for investigating the structure and diversity of soil microorganisms [12,13]. Du reported that the composition of the bacterial community in GM rice soil differs from that in non-GM rice soil [14]. Fazal found that the relative abundance of the nitrogen-fixing bacterium (*Sinorhizobium fredii*) increased in soil planted with GM soybean [15]. Yang observed that the relative abundances of rhizobia *Ensifer fredii* and *Bradyrhizobium elkanii* were significantly lower in GM soybean soil compared to non-GM soybean soil [16]. Despite its widespread use, there is an ongoing debate regarding the effect of glyphosate exposure on soil microbial communities. Li found that glyphosate can stimulate bacterial growth at high doses and tends to enhance bacterial diversity at lower doses [17]. Vázquez reported that glyphosate negatively affects soil fungal communities [18]. The impact of GM crops under glyphosate application is insufficiently understood.

China is the largest consumer and importer of soybeans in the world. Currently, China has approved multiple safety certificates for GM soybean cultivars. Some GM soybean cultivars have been planted for demonstrative purposes in certain parts of China. The alteration of the soil microbial community under actual field planting conditions is paramount in relation to GM soybean commercialization planting. The GM soybean cultivar used in this study was Maiyou 579, which was derived from the cp4-epsps and pat GM soybean DBN9004. By comparing it with the non-GM soybean, the impact of planting GM soybean on the soil bacterial and fungal communities has been investigated, and the changes in sensitive microbial species and their abundance were explored. The results reported herein enrich the environmental safety data and support the long-term safe use of herbicide-resistant soybeans.

## 2. Results

### 2.1. Species Composition and Relative Abundances

To analyze the taxonomic composition of soybean-associated bacterial and fungal communities, bacterial 16S rDNA sequences were aligned and assigned to 42 phyla, 133 classes, 321 orders, 513 families, 1009 genera, 2421 species, and 11,755 operational taxonomic units (OTUs). Fungal internal transcribed spacer (ITS) sequences were aligned and assigned to 15 phyla, 44 classes, 101 orders, 223 families, 454 genera, 765 species, and 2340 OTUs.

**Figure 1:**
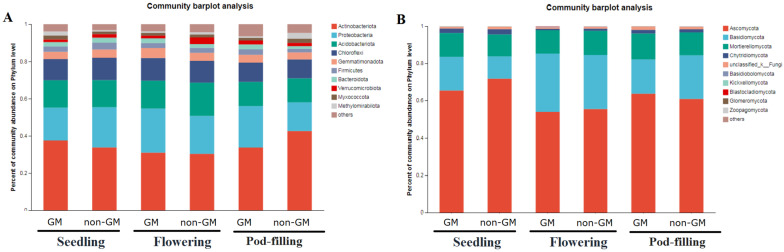
Bar plot showing phylum-level classification of bacterial community (**A**) and fungal community (**B**) at the seedling, flowering, and pod-filling stages.

The stacked bar plot was conducted to identify the most abundant bacterial communities both on phylum levels. Taxonomic groups with a relative abundance of under 1% in all samples were combined into others. The dominant phyla of the bacterial and fungal communities remained consistent between the GM and non-GM soybean crops throughout the entire crop cycle, albeit with the relative abundances of the dominant phyla in the soil community changing across different growth stages (Figure 1A). With respect to the bacterial community, the relative abundances of the top five phyla were Actinobacteria (30–42%), Proteobacteria (15–23%), Acidobacteriota (12–17%), Chloroflexi (10–13%), and Gemmatimonadota (3–5%). These five phyla collectively accounted for more than 80% of the total bacterial abundance. At the seedling stage, the relative abundance of Gemmatimonadota in GM soybeans was significantly lower than that at the flowering or pod-filling stages. No significant differences in the relative abundances of the dominant phyla were observed between GM and non-GM soybeans at the same growth stage (Kruskal–Wallis H test, *p* < 0.05).

As for the fungal community, Ascomycota was the most dominant phylum, with a relative abundance of over 50%, regardless of the growth stage or plant material (Figure 1B). The top five phyla in the soil fungal community were Ascomycota (relative abundance 53–71%), Basidiomycota (11–31%), Mortierellomycota (11–13%), Chytridiomycota (2–5%), and unclassified_k__Fungi (1–3%). These five phyla collectively accounted for more than 97% of the total abundance. The relative abundances of these five phyla changed at different growth stages, but there were no significant differences between the two soybean types at any stage (Kruskal–Wallis H test, *p* < 0.05).

The relative abundance of twenty-three bacterial genera exhibited significant differences in all growth stages between GM and non-GM soybeans by the Kruskal–Wallis test (Table 1). Actinobacteria and Proteobacteria each have six genera; Acidobacteria and Chloroflexiach each have three genera; meanwhile, Firmicutes, Desulfobacterota, and Gemmatimonadota each have three genera. Four genera were observed at the seedling stage, fourteen at flowering, and six at the pod-filling stage. Notably, the relative abundance of *Frankia* was significantly lower in the GM soybean at both the seedling and flowering stages. Conversely, *Aeromicrobium* showed higher relative abundance in the GM soybean at flowering but lower abundance at the pod-filling stage.

The relative abundance of four fungal genera from the Ascomycota phylum demonstrated significant differences throughout the entire crop cycle. One genera exhibited significant variations at the seedling stage, four at flowering, and two at the pod-filling stage. Among these, the relative abundance of *Thelebolus* showed significant differences during both the flowering and pod-filling stages, with higher abundances observed in GM soybeans compared to non-GM soybeans.

### 2.2. Alpha Diversity Analysis

The rarefaction curves for the observed OTU number and ACE almost reached the saturation plateau, indicating that the OTU coverage was sufficient to encompass enough detectable species in the soil microbial community and capture the diversity of soil microbial communities (Table 2). The differences in soil microbial alpha diversity (the Shannon, Simpson, and ACE indices) between GM and non-GM soybeans were examined at three growth stages. The bacterial ACE index did not exhibit significant differences between the two soybean soils at the same growth stage. However, at flowering, the bacterial Shannon index of non-GM soybeans was significantly higher than that of GM soybeans, whereas the Simpson index of non-GM soybeans was significantly lower than that of GM soybeans during the pod-filling stage. There were no significant differences in any of the three indices for soil fungal alpha diversity between GM and non-GM soybean soils.

### 2.3. Beta Diversity Analysis

**Figure 2:**
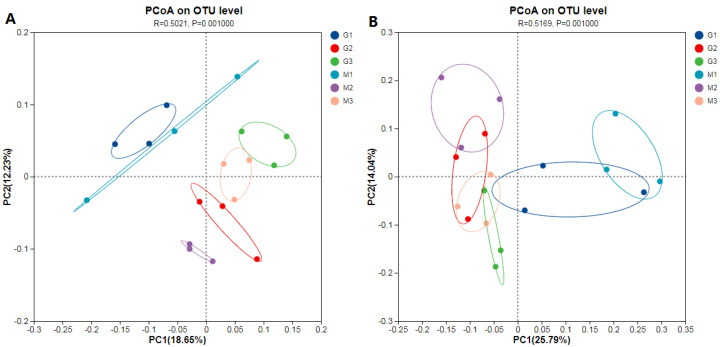
PCoA using the Bray–Curtis metric showed that the growth stage was the largest source of variance among rhizosphere soil microbial communities. (**A**): bacterial community; (**B**): fungal community; M: non-transgenic; 1: seedling stage; 2: flowering stage; 3: pod-filling stage.

Bacterial and fungal communities at the seedling, flowering, and pod-filling stages were analyzed using PcoA (Figure 2). When the three growth stages were examined together, the GM and non-GM soybean groups clustered together during the seedling stage, both in bacterial and fungal communities. This observation suggests that the soil community structure at the seedling stage significantly differed from that of the other two stages. The permutational multivariate analysis of variance using the Bray–Curtis metric revealed that growing stage caused the greatest difference between bacterial and fungal communities (ANOSIM: [bacteria: R = 0.50, *p* = 0.001; and fungi: R = 0.52, *p* = 0.001]).

The bacterial and fungal communities of GM and non-GM soybeans at the same growth stage were analyzed. The GM and non-GM soybeans were generally distinguished by principal components PC1 or PC2. However, there were no significant differences in bacterial or fungal communities between the two soybean soils (ANOSIM, *p* > 0.05). The different growth stages had significant impacts on the composition of the rhizosphere soil microbial community.

### 2.4. Nodule Quantity and Rhizobial Community

The number of nodules in the GM soybean roots was 60.13 ± 11.29 per plant, which was slightly higher than that of the non-GM soybean roots (56.33 ± 16.70), although the difference between the two plant types was not significant.

Eleven genera of rhizobia were detected in both GM and non-GM soil bacterial communities (Table 3). *Rhizobium*, *Neorhizobium*, and *Parrhizobium* were grouped together, as they could not be distinguished based on the 16S rRNA fragments. *Phyllobacterium, Shinella,* and *Azorhizobium* were not detected at any of the three growth stages under study. The relative abundance of *Bradyrhizobium* was significantly lower in those that were GM than in the non-GM soybeans. However, the relative abundance of *Bradyrhizobium* was restored at flowering and pod-filling stages, and there was no significant difference between the two treatments.

## 3. Discussion

Numerous studies indicate that the impact of transgenic crops on soil ecosystems is both temporary and slight. Moreover, various factors (including site location, climate conditions, and management systems) exert a more significant influence on soil ecosystems than transgenic crops [19]. Soil microorganisms play an important role in soil biochemical processes, including soil nutrient transformation, organic matter decomposition, humus formation, and pollutant biodegradation [20]. Moreover, changes in soil microbial community diversity can reflect the status of the soil environment [21]. The growth status of GM plants and the introduced exogenous genes can change the composition and quantity of root exudates and their secretion, which may affect the growth, community diversity, and function of soil microorganisms [22,23,24]. Huang reported that Bt protein from transgenic Bt rice may be an intriguing factor for bacterial diversity variations [25]. Changes in specific soil properties, such as pH, texture, and nitrogen availability, also lead to changes in microorganism assemblages [26]. To minimize interference from soil nutrients and other factors on the experimental results, both the transgenic and control treatments were placed within the same plot. No significant differences in soil nutrients were observed between the two treatments (Table 4). This study analyzed the differences in rhizosphere soil bacterial and fungal communities between herbicide-tolerant GM soybeans and non-GM soybeans using HiSeq sequencing. The alpha diversity results indicated that GM soybeans did not consistently affect the bacterial and fungal communities in soybean roots. The results showed that GM soybeans altered the relative abundance of a few soil microorganisms. Compared with non-GM soybeans, there were some differences in the composition and structure of bacteria and fungi communities in the GM soybean rhizosphere, although these differences were not significant.

### 3.1. Microbial Community Structure

Compared to non-GM soybeans, there were no significant differences in the bacterial or fungal community composition at the OUT level (PCoA). However, there were significant differences in the alpha diversity indices of the microbial communities between GM and non-GM soybeans at specific growth stages. In particular, the Shannon index of the rhizosphere soil bacterial community of GM soybean plants was significantly higher than that of non-GM soybean plants, indicating that planting GM soybeans can improve the diversity of the rhizosphere soil bacterial community compared with that of the non-GM soybean plants. However, this difference was not observed across all three different growth stages. Additionally, there were no significant differences in the three indices of rhizosphere soil fungal alpha diversity (the Shannon, Simpson, or Ace index) between the GM and non-GM soybean plants, consistent with Wang’s report [27]. In contrast, Shen reported that glyphosate-resistant soybean plants significantly increased the abundance and diversity of rhizosphere soil bacterial communities at the pod-filling stage [28]. Previous research has suggested that there were no or minor changes in microbial communities, and the differences between GM and non-GM plants were temporary [15,29]. Lu reported that the observed differences in the Shannon index between GM crops and their corresponding counterparts may be caused by differences in rhizosphere metabolites and agronomic management practices [30]. The inconsistent effects of GM crops on the rhizosphere community could possibly be attributed to soil heterogeneity and a lack of effective controls.

### 3.2. Relative Abundance of Important Functional Groups

Plants can enhance their resistance against biotic and abiotic stresses by affecting the assembly of rhizosphere microorganisms, particularly by enriching populations of plant growth-promoting bacteria and fungi [31]. Newman found that, compared with the control, the relative abundance of Proteobacteria increased upon glyphosate treatment, with Gamma proteobacteria showing the most significant increase [32]. Babujia reported that GM soybeans increased the abundance of rhizosphere Proteobacteria, Firmicutes, and Chlorophyta while reducing the abundance of Actinobacteria and Acidobacteria [33]. Meanwhile, our results showed that Proteobacteria, Acidobacteria, and Bacteroidetes were the dominant bacterial groups in both soybean treatments, and there was no significant difference in the relative abundance of the main bacterial groups between them for the same period. Barriuso found no significant difference in rhizosphere bacterial abundance or richness between Bt transgenic maize and the parental types, which had little effect on the bacterial community structure, whereas the plant growth stage or environmental factors may have a more significant effect on the microbial community [34]. Griffiths found that GM maize had a certain impact on soil microorganisms but was seemingly more influenced by the soil type and insecticide [35]. In our study, different growth stages also had an exclusionary effect on bacteria and fungi recruitment. In the flowering stage, GM soybean caused an enrichment of *Aeromicrobium* whereas *Frankia* was more abundant. Similarly, non-GM soybeans had a substantial effect on *Striatibotrys* abundance during the seedling stage. The genus *Thelebolus* is a prevalent fungus found in soil. Bai reported that continuous soybean cropping affects fungal diversity in rhizosphere soils and significantly increases the abundance of *Thelebolus* [36]. This genus has garnered considerable attention due to its capability to grow and produce enzymes that decompose straw under low-temperature conditions [37,38]. Our research field was located in Inner Mongolia, China, where the prolonged duration of low temperatures hampers straw degradation in the soil. This study reveals that the relative abundance of *Thelebolus* significantly increased in genetically modified soybean treatments during the later stages of growth. However, whether this enhancement is beneficial for straw degradation remains to be further investigated. Our results suggested that GM soybeans and glyphosate application have a minimal effect on the composition of the soybean microbial community but have no impact on the structure of the rhizosphere microbial community.

### 3.3. Nodulation

Root nodules are specific symbiotic structures formed between leguminous plants and rhizobia, involving complex signal recognition, gene expression regulation, and energy exchange [39]. There are approximately 100 species belonging to 19 genera of bacteria that can form symbiotic root nodules with leguminous plants. These rhizobia are predominantly classified under the α- and β-proteobacteria [40]. Lu reported that ten major symbiotic nitrogen-fixing bacterial genera were detected, but not all of these genera were found in both GM and non-GM soybean samples [41]. This study detected 11 rhizobial genera that were present in three growth stages of both genetically modified (GM) and non-GM soybean soils. Our results indicated that the predominant symbiotic nitrogen-fixing bacterial genera were not significantly impacted by genetically modified (GM) soybeans throughout the growing stage.

*Bradyrhizobium* and *Sinorhizobium* have been reported as the main rhizobia that can nodulate in soybean plants. Rhizobia compete with each other in soils with varying pH levels. In acidic soils, *Bradyrhizobium* is predominant in nodules of the soybean; conversely, in alkaline soils, *Sinorhizobium* is predominant in soybean nodules [41]. The growth of *B. japonicum* was inhibited by glyphosate [42]. Yang reported that the relative abundances of rhizobia *Ensifer fredii* and *Bradyrhizobium elkanii* were significantly different between GM and non-GM soybeans [43]. Our study found that the relative abundance of *Bradyrhizobium* in GM soybean soil at the seedling stage was significantly lower than that in non-GM soybeans, presumably owing to the presence of glyphosate and its metabolites in the soil. The relative abundance of *Bradyrhizobium* was restored at flowering and pod-filling stages, and there was no significant difference between GM and non-GM soybean soils. Powell found that glyphosate had adverse effects on the establishment and function of root nodule symbionts [44]. Han found that bacterial microbiota play a crucial role in shaping rhizobial–host interactions in soybeans, and the rhizosphere community composition of soybeans varied significantly in different soils [45]. Our results showed that neither the GM soybean nor glyphosate had any significant effect on the number of soybean nodules. However, we have only counted the total number of root nodules and have not investigated the efficiency of nitrogen fixation. Thus, the impact of a low relative abundance of *Bradyrhizobium* in seedlings on nitrogen fixation should be continuously monitored.

## 4. Materials and Methods

### 4.1. Experimental Design and Soil Sampling

In this study, non-GM soybeans (Heifeng 50) and GM soybeans (Mianyou 579) carrying the *cp4-epsps* and *pat* genes were used as experimental plant material. The study site was a field located in Keyouqian Banner, Xing’an League, Inner Mongolia, China. GM and non-GM soybean seeds were planted adjacent to each other in the experimental plot at a distance of 5 m, each covering an area of approximately 0.4 hectares. The two plant types experienced similar basic soil physicochemical properties (Table 4). Soybean seeds were planted in late May 2023. In mid-June, glyphosate (2.5 L/ha, 43%) was applied to GM soybeans, whereas herbicide mixtures (Quinquefolin 80 g/ha, bentazone 120 g/ha, formesafen 550 g/ha) were applied to the non-GM soybean. Other agronomic management practices were the same for both plant types.

Soil samples were collected at the seedling (June 26), flowering (July 22), and pod-filling stages (August 20). Three replicates were collected for each sampling point in the field, following an S-shaped walking path. Soil samples were deliberately avoided from the areas near the edges of the experimental site to prevent cross-contamination between treatments. Soil samples were collected around the roots at depths of 5–20 cm and stored at −80 °C for DNA extraction. Three replicates for each of the three developmental stages yielded 18 samples. The bulk soils used to determine the characteristics were all filtered through a 50 μm mesh sieve after natural air drying. The methods used for the determination of soil nutrients (Table 4) are listed as follows: Organic matter was determined using the potassium dichromate volumetric method; total nitrogen was determined using the semi-micro Kjeldahl method; total phosphorus was determined using sulfuric–perchloric acid digestion and molybdenum–antimony colorimetry; alkali hydrolyzed nitrogen was determined using the alkaline hydrolysis diffusion method; available phosphorus was determined using the sodium bicarbonate extraction molybdenum–antimony anti-colorimetric method; the available potassium was determined using the ammonium acetate extraction-flame photometry method; and at the pod-filling stage, the numbers of nodules per plant were determined. A total of 20 soybean plants were investigated for each treatment.

### 4.2. Sample Preparation and PCR Amplification

Soil DNA was extracted using the Fast DNA^®^ SPIN Kit (MP Biomedicals, Santa Ana, CA, USA). The quality and concentration of DNA were determined by 1.0% agarose gel electrophoresis and a NanoDrop1000 spectrophotometer (Nanodrop, Wilmington, NC, USA) and kept at -80 °C prior to further use. PCR amplification of the bacterial 16S rDNA V3-4 region was performed using the universal primer pair 338F/806R (338F: ACTCCTACGGGAGGCAGCAG, 806R: GGACTACHVGGGTWTCTAAT), and amplification of the fungal ITS region was performed using the universal primer pair ITS1F/ITS1R (ITS1F: CTTGGTCATTTAGAGGAAGTAA, ITS1R: GCTGCGTTCTTCATCGATGC). The PCR reaction mixture included 5 μL of 5 × Fast Pfu buffer, 2.5 μL of 2.5 mM dNTPs, 1 μL of each primer (5 μM), 0.5 μL of Fast Pfu polymerase, 10 ng of extracted DNA, and ddH2O to a final volume of 25 µL. The PCR amplification cycling conditions were as follows: initial denaturation at 95 °C for 3 min, followed by 28 cycles of denaturing at 95 °C for 30 s, annealing at 55 °C for 30 s, extension at 72 °C for 45 s, single extension at 72 °C for 10 min, and end at 4 °C. The PCR products were extracted from a 2% agarose gel and purified using a PCR Clean-Up Kit (Axygen, San Francisco, CA, USA).

### 4.3. Illumina Hiseq Sequencing and Metagenomic Data Analysis

Purified amplicons were pooled in equimolar amounts, and paired-end sequenced on an Illumina PE300 platform (Illumina, San Diego, CA, USA) according to the standard protocols of Majorbio Bio-Pharm Technology Co. Ltd. (Shanghai, China).

After demultiplexing, the resulting sequences were quality-filtered using fastp (0.19.6) and merged using FLASH (v1.2.11). High-quality sequences were denoised using the DADA2 plugin in the Qiime2 (version 2020.2) pipeline with the recommended parameters, which allows single-nucleotide resolution based on the error profiles within samples. To minimize the effects of the sequencing depth on the alpha and beta diversity indices, the number of sequences from each sample was rarefied. The taxonomy of each OTU representative sequence was analyzed by RDP Classifier version 2.2 against the Silva v138 16S rRNA gene and Unite 8.0/ITS fungi databases using a confidence threshold of 0.7.

Bioinformatics analysis of the soil microbiota was performed using the Majorbio Cloud platform (https://cloud.majorbio.com).Based on the OTU information, rarefaction curves and alpha diversity indices were calculated. Comparisons of taxonomic data at the phylum and genus levels were tested using the Kruskal–Wallis test with a post hoc Tukey’s HSD test using the stats package in R (v3.3.1). Statistical significance was accepted as *p*  < 0.05. Rarefaction curve construction, as well as alpha diversity indices (ACE, Shannon, and Simpson), were calculated on a rarified dataset (30,000 sequences) using Mothur (v1.30.2). Alpha diversity indices were compared with the Kruskal–Wallis test using the *stats* package in R. Similarities among the microbial communities in different samples were determined by principal coordinate analysis (PCoA) based on Bray–Curtis dissimilarity using the *vegan* package in R (v3.3.1). The PERMANOVA test (999 permutations) based on the Bray–Curtis distance was used to test for significant differences among the microbial communities of different samples using the *adonis* package in R (v3.3.1). An analysis of similarities (ANOSIM) based on the Bray–Curtis distance was carried out to test for significant differences among the microbial communities of different samples using the vegan package in R.

## 5. Conclusions

The hypothesis of this study posits that genetically modified soybeans, along with their agronomic practices, induce changes in soil microbial community diversity and the abundance of key species compared to conventional cultivation. High-throughput sequencing was used to evaluate the potential effects of GM soybeans with glyphosate application on soil microbial microbial diversity. At the phylum level, bacterial and fungal community compositions were consistent between glyphosate-tolerant and non-GM soybean soils at the same stage. The PCoA analysis indicated that GM soybeans influenced soil microorganisms, but there were no significant differences in the community structure. The growth stage was found to have a greater impact on soil microbes compared to GM soybeans. Neither GM soybeans nor glyphosate significantly altered the number of root nodules in soybeans, but they significantly reduced the relative abundance of *Bradyrhizobium* in rhizosphere soil at the seedling stage. Meanwhile, the relative abundance of *Frankia* in the rhizosphere soil of GM soybeans was significantly lower than in that of non-GM soybeans at the seedling and flowering stages. At the flowering and pod-filling stages, the relative abundance of *Thelebolus* was significantly higher in the rhizosphere of those GM than in the rhizosphere of non-GM soybean plants. The abundance and ecological functions of these species should be continuously monitored.

## Figures and Tables

**Table 1:** Differentially abundant genera identified from the 16S and ITS databases.

Classification	Phylum	Genus	Seedling	Florescence	Pod Filling
Bacteria	Actinobacteriota	*Aeromicrobium*	0.83	0.04 *	0.02 *
		*Jatrophihabitans*	0.21	0.03 *	0.41
		*Frankia*	0.17	0.03 *	0.04 *
	Proteobacteria	*Bradyrhizobium*	0.04 *	0.11	0.16
		*Reyranella*	0.44	0.04 *	0.22
		*Ramlibacter*	0.87	0.03 *	0.42
		*Nordella*	0.04 *	0.11	0.16
		*MND1*	0.02 *	0.56	0.29
		*Mesorhizobium*	0.03 *	0.10	0.18
	Acidobacteriota	g__norank_f__norank_o__Subgroup_7	0.07	0.08	0.03 *
		Frankia	0.04 *	0.03 *	0.89
		g__norank_f__Vicinamibacteraceae	0.22	0.03 *	0.70
		g__Subgroup_10	0.97	0.04 *	0.06
	Chloroflexi	g__norank_f__norank_o__SBR1031	0.49	0.05 *	0.14
		g__norank_f__Anaerolineaceae	0.25	0.03 *	0.87
		g__norank_f__JG30-KF-CM45	0.34	0.37	0.15
		g__norank_f__A4b	0.10	0.04 *	0.17
	Firmicutes	*Bacillus*	0.9	0.13	0.10
	Desulfobacterota	g__norank_f__norank_o__norank_c__norank_p__Desulfobacterota	0.33	0.21	0.04 *
	Gemmatimonadota	*Gemmatimonas*	0.06	0.33	0.03 *
Fungi	Ascomycota	*Striatibotrys*	0.03 *	0.25	0.07
		*Thelebolus*	0.06	0.05 *	0.08 *
		g__unclassified_c__Dothideomycetes	0.70	0.03 *	0.13
		*Penicillium*	0.38	0.03 *	0.11

* Significant difference at 0.05 level. Kruskal–Wallis test.

**Table 2:** Alpha diversity of microbial communities at the OUT level.

Index	Classification	Treatments	Seedling	Florescence	Pod Filling
Shannon	Bacteria	GM	6.74 ± 0.013	6.63 ± 0.033 *	6.58 ± 0.023
		Non-GM	6.58 ± 0.13	6.74 ± 0.035	6.71 ± 0.046
	Fungi	GM	3.87 ± 0.26	3.69 ± 0.12	3.88 ± 0.37
		Non-GM	3.68 ± 0.15	3.67 ± 0.18	3.91 ± 0.17
Simpson	Bacteria	GM	0.0043 ± 0.00051	0.0056 ± 0.00060	0.0068 ± 0.00044 *
		Non-GM	0.0049 ± 0.00033	0.0048 ± 0.00022	0.0049 ± 0.00075
	Fungi	GM	0.052 ± 0.014	00069 ± 0.017	0.055 ± 0.021
		Non-GM	0.068 ± 0.014	0.059 ± 0.013	0. 050 ± 0.0099
Ace	Bacteria	GM	5219.4 ± 66.64	5184.7 ± 796.54	4628.5 ± 126.92
		Non-GM	4611.4 ± 314.09	5237.1 ± 131.51	4931.5 ± 154.68
	Fungi	GM	754.27 ± 46.47	707.26 ± 50.48	811.49 ± 75.16
		Non-GM	705.36 ± 97.87	680.39 ± 41.70	723.96 ± 24.08

* Significant difference at 0.05 level. (*p* < 0.05, Kruskal–Wallis test).

**Table 3:** Relative abundance of rhizobial communities at genus level.

Genus	Seedling	Florescence	Pod-Filling
*Bradyrhizobium*	0.04 *	0.33	0.63
*Ochrobactrum*	0.33	0.97	0.06
*Devosia*	0.50	0.14	0.78
*Methylobacterium*	0.62	0.40	0.83
*Microvirga*	0.32	0.23	0.68
*Mesorhizobium*	0.03	0.13	0.18
*Rhizobium-Neorhizobium Pararhizobium* ^a^	0.48	0.11	0.80
*Sinorhizobium/Ensifer*	0.46	0.09	0.40
*Burkholderia*	0.14	0.13	0.74
*Cupriavidus*	0.68	0.40	0.18
*Pseudomonas*	0.25	0.72	0.13

* Significant difference at 0.05 level (*p* < 0.05, Kruskal–Wallis test). ^a^ *Rhizobium*, *Neorhizobium*, and *Parrhizobium* were grouped together, as they could not be distinguished based on the 16S rRNA fragments.

**Table 4:** Soil characteristics.

Treatments	Organic Matter g/kg	Total Nitrogen g/kg	Total Potassium g/kg	Total Phosphorus g/kg	Alkali Hydrolyzed Nitrogen mg/kg	Available Phosphorus mg/kg	Available Potassium mg/kg
GM	16.77 ± 1.24	1.19 ± 0.04	10.72 ± 0.13	1.28 ± 0.34	98.19 ± 11.96	21.24 ± 4.21	8.01 ± 1.14
Non-GM	18.24 ± 2.11	1.11 ± 0.17	11.70 ± 0.11	1.04 ± 0.14	101.24 ± 5.24	18.74 ± 3.41	7.55 ± 0.97

## Data Availability

The original contributions presented in the study are included in the article, further inquiries can be directed to the corresponding author.

## References

[1] (2024). AgbioInvestor. Global GM Crop Area 2023 Review. https://gm.agbioinvestor.com/downloads.

[2] Schmalenberger A., Tebbe C.C. (2002). Bacterial community composition in the rhizosphere of a transgenic,herbicide-resistant maize (*Zea mays*) and comparison to its non-transgenic cultivar Bosphore. FEMS Microbiol. Ecol..

[3] Brookes G., Barfoot P. (2014). Economic impact of GM crops: The global income and production effects 1996–2012. Gm Crops Food.

[4] George T.S., Richardson A.E., Li S.S., Gregory P.J., Daniell T.J. (2009). Extracellular release of a heterologous phytase from roots of transgenic plants: Does manipulation of rhizosphere biochemistry impact microbial community structure?. FEMS Microbiol. Ecol..

[5] Mocali S., Dentice A., Marcucci A., Benedetti A. (2009). The impact of post-harvest treatments of transgenic eggplant residues on soil quality and microbial diversity. Ultrasound Med. Biol..

[6] Duke S.O. (2005). Taking stock of herbicide-resistant crops ten years after introduction. Pest Manag. Sci..

[7] Landrigan P.J., Belpoggi F. (2018). The need for independent research on the health effects of glyphosate-based herbicides. Environ. Health.

[8] Zhou Y., Yang Z., Liu J., Li X., Wang X., Dai C., Zhang T., Carrión V.J., Wei Z., Cao F. (2023). Crop rotation and native microbiome inoculation restore soil capacity to suppress a root disease. Nat. Commun..

[9] Nadarajah K., Abdul R. (2021). Plant-microbe interaction: Aboveground to belowground, from the good to the bad. Int. J. Mol. Sci..

[10] Li X.G., Wei Q., Liu B., Alam M.S., Wang X.X., Shen W., Han Z.M. (2013). Root exudates of transgenic cotton and their effects on *Fusarium oxysporum*. Front. Biosci. (Landmark Ed.).

[11] Christ B., Hochstrasser R., Guyer L., Francisco R., Aubry S., Hörtensteiner S., Weng J.K. (2017). Non-specific activities of the major herbicide-resistance gene BAR. Nat. Plants.

[12] Rousk J., Brookes P.C., Bååth E. (2010). The microbial PLFA composition as affected by pH in an arable soil. Soil Biol. Biochem..

[13] Lucas J.A., García-Villaraco A., Ramos B., García-Cristobal J., Algar E., Gutierrez-Mañero J. (2013). Structural and functional study in the rhizosphere of *Oryza sativa* L. plants growing under biotic and abiotic stress. J. Appl. Microbiol..

[14] Du L., Wang Y., Shan Z., Shen X., Wang F., Su J. (2021). Comprehensive analysis of SUSIBA2 rice: The low-methane trait and associated changes in soil carbon and microbial communities. Sci Total Environ..

[15] Fazal A., Yang M., Wang X., Lu Y., Yao W., Luo F., Han M., Song Y., Cai J., Yin T. (2023). Discrepancies in rhizobacterial assembly caused by glyphosate application and herbicide-tolerant soybean Co-expressing GAT and EPSPS. J. Hazard. Mater..

[16] Yang M., Wen Z., Fazal A., Hua X., Xu X., Yin T., Qi J., Yang R., Lu G., Hong Z. (2020). Impact of a G2-EPSPS & GAT Dual transgenic glyphosate-resistant soybean line on the soil microbial community under field conditions affected by glyphosate application. Microbes Environ..

[17] Li W., Wang K., Wang P., Yang P., Xu S., Tong J., Zhang Y., Yang Y., Han L., Ye M. (2025). Impact of glyphosate on soil bacterial communities and degradation mechanisms in large-leaf tea plantations. J. Hazard. Mater..

[18] Vázquez M.B., Moreno M.V., Amodeo M.R., Bianchinotti M.V. (2021). Effects of glyphosate on soil fungal communities: A field study. Rev. Argent. Microbiol..

[19] Lebedev V., Lebedeva T., Tikhonova E., Shestibratov K. (2022). Assessing Impacts of Transgenic Plants on Soil Using Functional Indicators: Twenty Years of Research and Perspectives. Plants.

[20] Liu L., Cheng L., Liu L., Cheng L., Liu K., Yu T., Liu Q., Gong Z., Cai Z., Liu J. (2023). Transgenic soybean of GsMYB10 shapes rhizosphere microbes to promote resistance to aluminum (Al) toxicity. J. Hazard. Mater..

[21] Liu W., Lu H.H., Wu W., Wei Q.K., Chen Y.X., Thies J.E. (2008). Transgenic Bt rice does not affect enzyme activities and microbial composition in the rhizosphere during crop development. Soil Biol. Biochem..

[22] Khan M.S., Sadat S.U., Jan A., Munir I. (2017). Impact of Transgenic *Brassica napus* Harboring the Antifungal Synthetic Chitinase (NiC) Gene on Rhizosphere Microbial Diversity and Enzyme Activities. Front. Plant Sci..

[23] Han C., Liu B., Zhong W. (2018). Effects of transgenic Bt rice on the active rhizospheric methanogenic archaealcommunity as revealed by DNA-based stable isotope probing. J. Appl. Microbiol..

[24] Yang S., Wang G., Niu M., Zhang H., Ma J., Qu C., Liu G. (2024). Impacts of AlaAT3 transgenic poplar on rhizosphere soil chemical properties, enzyme activity, bacterial community, and metabolites under two nitrogen conditions. GM Crops Food.

[25] Huang Q., Zhang Y., Tan Y., Kong H., Cao Y., Wang J., Yin G., Guo A. (2024). *Bt*-Modified Transgenic Rice May Shift the Composition and Diversity of Rhizosphere Microbiota. Plants.

[26] Labouyrie M., Ballabio C., Romero F., Panagos P., Jones A., Schmid M.W., Mikryukov V., Dulya O., Tedersoo L., Bahram M. (2023). Patterns in soil microbial diversity across Europe. Nat. Commun..

[27] Wang G., Yang S., Feng S., Zhao G., He X., Han X. (2024). Impact of glyphosate on the rhizosphere microbial communities of a double-transgenic maize line D105. Front. Sustain. Food Syst..

[28] Shen B., Hong X., Cao Y.P., Han C., Liu B., Zhong W.H. (2018). Effects of glyphosate-resistant transgenic soybean on soil rhizospheric bacteria and rhizobia. Chin. J. Appl. Ecol..

[29] He M., Zhang J., Shen L., Xu L., Luo W., Li D., Zhai N., Zhao J., Long Y., Pei X. (2019). High-throughput sequencing analysis of microbial community diversity in response to indica and japonica bar-transgenic rice paddy soils. PLoS ONE.

[30] Lu G.H., Tang C.Y., Hua X.M., Cheng J., Wang G.H., Zhu Y.L., Zhang L.Y., Shou H.X., Qi J.L., Yang Y.H. (2018). Effects of an EPSPS-transgenic soybean line ZUTS31 on root-associated bacterial communities during field growth. PLoS ONE.

[31] Pieterse C.M., Zamioudis C., Berendsen R.L., Weller D.M., Van Wees S.C., Bakker P.A. (2014). Induced systemic resistance by beneficial microbes. Annu. Rev. Phytopathol..

[32] Newman M.M., Hoilett N., Lorenz N., Dick R.P., Liles M.R., Ramsier C., Kloepper J.W. (2016). Glyphosate effects on soil rhizosphere-associated bacterial communities. Sci. Total Environ..

[33] Babujia L.C., Silva A.P., Nakatani A.S., Cantão M.E., Vasconcelos A.T., Visentainer J.V., Hungria M. (2016). Impact of long-term cropping of glyphosate-resistant transgenic soybean [*Glycine max* (L.) Merr.] on soil microbiome. Transgenic. Res..

[34] Barriuso J., Valverde J.R., Mellado R.P. (2012). Effect of Cry1Ab protein on rhizobacterial communities of Bt-maize over a four-year cultivation period. PLoS ONE.

[35] Griffiths B.S., Caul S., Thompson J., Birch A.N., Scrimgeour C., Cortet J., Foggo A., Hackett C.A., Krogh P.H. (2016). Soil Microbial and Faunal Community Responses to Maize and Insecticide in Two Soils. J. Appl. Microbiol..

[36] Bai L., Cui J., Jie W., Cai B. (2015). Analysis of the community compositions of rhizosphere fungi in soybeans continuous cropping fields. Microbiol. Res..

[37] Mi Z., Su J., Yu L., Zhang T. (2024). Comparative mitochondrial genomics of Thelebolaceae in Antarctica: Insights into their extremophilic adaptations and evolutionary dynamics. IMA Fungus.

[38] da Silva M.K., Barreto D.L.C., Vieira R., Neto A.A., de Oliveira F.S., Convey P., Rosa C.A., Duarte A.W.F., Rosa L.H. (2024). Diversity and enzymatic, biosurfactant and phytotoxic activities of culturable Ascomycota fungi present in marine sediments obtained near the South Shetland Islands, maritime Antarctica. Extremophiles.

[39] Tripathi B.M., Kim M., Lai-Hoe A., Shukor N.A., Rahim R.A., Go R., Adams J.M. (2013). pH dominates variation in tropical soil archaeal diversity and community structure. FEMS Microbiol. Ecol..

[40] Kuzmanović N., Fagorzi C., Mengoni A., Lassalle F., diCenzo G.C. (2022). Taxonomy of Rhizobiaceae revisited: Proposal of a new framework for genus delimitation. Int. J. Syst. Evol. Microbiol..

[41] Lu G.H., Zhu Y.L., Kong L.R., Cheng J., Tang C.Y., Hua X.M., Meng F.F., Pang Y.J., Yang R.W., Qi J.L. (2017). Impact of a glyphosate-tolerant soybean line on the rhizobacteria, revealed by illumina miseq. J. Microbiol. Biotechnol..

[42] Zablotowicz R.M., Reddy K.N. (2004). Impact of glyphosate on the *Bradyrhizobium japonicum* symbiosis with glyphosate-resistant transgenic soybean: A minireview. J. Environ. Qual..

[43] Yang Y., Wen C., Hao C., Fazal A., Liao F., Luo F., Yao W.C., Yin T., Yang R., Qi J. (2021). Differential assembly and shifts of the rhizosphere bacterial community by a dual transgenic glyphosate-tolerant soybean line with and without glyphosate application. Horticulturae.

[44] Powell J.R., Campbell R.G., Dunfield K.E., Gulden R.H., Antunes P.M. (2009). Effect of glyphosate on the tripartite symbiosis formed by *Glomus intraradices*, *Bradyrhizobium japonicum*, and genetically modified soybean. Appl. Soil Ecol..

[45] Han Q., Ma Q., Chen Y., Tian B., Xu L., Bai Y., Chen W., Li X. (2020). Variation in rhizosphere microbial communities and its association with the symbiotic efficiency of rhizobia in soybean. ISME J..

